# Shape and Site Dependent *in Vivo* Degradation of Mg-Zn Pins in Rabbit Femoral Condyle

**DOI:** 10.3390/ijms15022959

**Published:** 2014-02-20

**Authors:** Pei Han, Moyan Tan, Shaoxiang Zhang, Weiping Ji, Jianan Li, Xiaonong Zhang, Changli Zhao, Yufeng Zheng, Yimin Chai

**Affiliations:** 1 Orthopaedic Department, Shanghai Jiao Tong University Affiliated Sixth People’s Hospital, Shanghai 200233, China; E-Mails: hanpei_cn@163.com (P.H.); jiweiping@126.com (W.J.); 2 College of Sciences, Shanghai Institute of Technology, Shanghai 201418, China; E-Mail: moyantan@163.com; 3 Suzhou Origin Medical Technology Co. Ltd., Suzhou 215513, China; E-Mail: zhangsx@126.com; 4 State Key Laboratory of Metal Matrix Composites, Institute of Biomedical Materials, School of Materials Science and Engineering, Shanghai Jiao Tong University, Shanghai 200240, China; E-Mails: lijianan@126.com (J.L.); zhangxiaonong@126.com (X.Z.); 5 Department of Advanced Materials and Nanotechnology, College of Engineering, Peking University, Beijing 100871, China; E-Mail: yfzheng@pku.edu.cn

**Keywords:** magnesium alloy, shape dependent, site dependent, *in vivo* degradation

## Abstract

A type of specially designed pin model of Mg-Zn alloy was implanted into the full thickness of lesions of New Zealand rabbits’ femoral condyles. The recovery progress, outer surface healing and *in vivo* degradation were characterized by various methods including radiographs, Micro-CT scan with surface rendering, SEM (scanning electron microscope) with EDX (Energy Dispersive X-ray analysis) and so on. The *in vivo* results suggested that a few but not sufficient bridges for holding force were formed between the bone and the implant if there was a preexisting gap between them. The rapid degradation of the implantation in the condyle would result in the appearance of cavities. Morphological evaluation of the specially designed pins indicated that the cusp was the most vulnerable part during degradation. Furthermore, different implantation sites with distinct components and biological functions can lead to different degradation rates of Mg-Zn alloy. The rate of Mg-Zn alloy decreases in the following order: implantation into soft tissue, less trabecular bone, more trabecular bone, and cortical bone. Because of the complexities of *in vivo* degradation, it is necessary for the design of biomedical Mg-Zn devices to take into consideration the implantation sites used in clinics.

## 1. Introduction

In the investigation of biodegradable Mg based implants, it was found that the physiological parameters in the environment have different effects on their degradation behavior [1–3]. The research of screws made from AZ31 magnesium alloy inserted into the hip bone of sheep showed that the screw threads located in the bone had slower degradation rates than the screw heads covered by soft tissue [4]. Moreover, the degradation of Mg-Mn-Zn and Mg-Ca alloy was faster in medullary cavity (within bone marrow) than in compact bone and spongy areas in *in vivo* experiment on rats and New Zealand white rabbits [5–8]. Thus, the degradation can be influenced by the implantation site. However, the influence of implantation site for Mg based implants has not been investigated in detail.

The femoral condyle which is composed of marrow, spongy bone, compact bone and cartilage was chosen as the implantation site. There are two kinds of lesions for the condyle site. One is “full thickness defect” which means that the lesion penetrates the subchondral bone. The other is called “partial thickness lesion” which means the defect does not reach the subchondral bone. The repair of both of them remains a current clinical problem. In this study, the full thickness defect was created to make as many kinds of environment as possible for the biomaterials. The main focus of this study is to analyze the biodegradation behavior of Mg-Zn alloy with simulated device shapes in different implantation sites. The various *in vivo* degradation behaviors affected by these factors were investigated and compared. The overall goal of this study is to provide guidance for the design of biodegradable Mg implants for clinic use.

## 2. Results

### 2.1. Gross Observation on the Outer Surface of the Condyle

**Figure 1.**
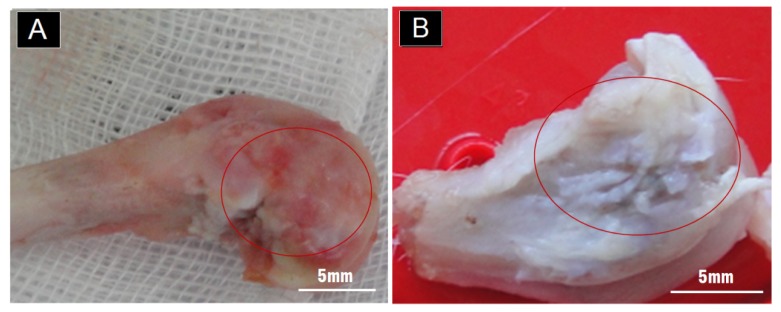
(**A**) The macro-photos of the outer surface after one month. Red circles indicated the implantation sites; (**B**) The macro-photos of the outer surface after three months. Red circles indicated the implantation sites.

The self-repair of the hyaline cartilage was unclear grossly (Figure 1), though there was no obvious hole on the smooth surface. The defects were successfully self-sealed after one month.

### 2.2. The Degradation Inside the Condyles Evaluated by Micro-CT

**Figure 2.**
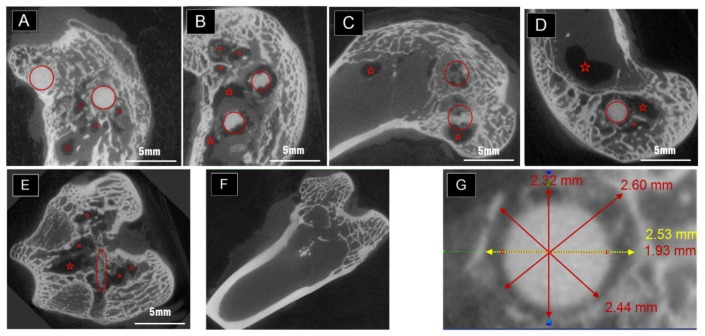
(**A**) Selected scanning planes after being examined by micro-CT one month post-operation with two pins in the condyle; (**B**) Selected scanning planes three months post-operation with two pins in the condyle; (**C**) Selected scanning planes six months post-operation with two pins in the condyle; (**D**,**E**) Selected scanning planes three months post-operation with one pin in the condyle; (**F**) Selected scanning planes three months post-operation for the condyle which only had drilled holes instead of biomaterials. (Red circles indicated the implantations approximately while the red stars located the sites of cavities); (**G**) Typical scanning plane of the “neck” part and measurement taken by the CT software after one month implantation.

The inside evaluation in the femoral condyle was characterized by Micro-CT, which can display gray level according to the structural density. The degradation behavior can be seen in Figure 2A–C, which was selected from the samples with two implants positioned in one condyle at one, three and six months post-operation. The red circles roughly indicated the position of the implantations. Meanwhile, the red stars in the pictures marked the darkest sites that represent cavities in the condyles. When there was only one pin placed in the rabbit, the cavities still existed as in Figure 2D,E. It is hard to tell the differences between the sizes of the cavities that belong to one-pin and two-pin implantation at the same time point. However, after six months, the size of the cavities decreased (see Figure 2C in comparison with Figure 2A,B after plane by plane general comparison (data not shown)). The normal healing progress was completed in Figure 2F. Bone marrow and new bone formation filled up the defects created by Kirschner pins during surgery in three months. No cavities were left in the samples which belong to the control group without Mg-Zn alloy as shown in Figure 2F.

### 2.3. The Degradation Evaluated by SEM and EDX

**Figure 3.**
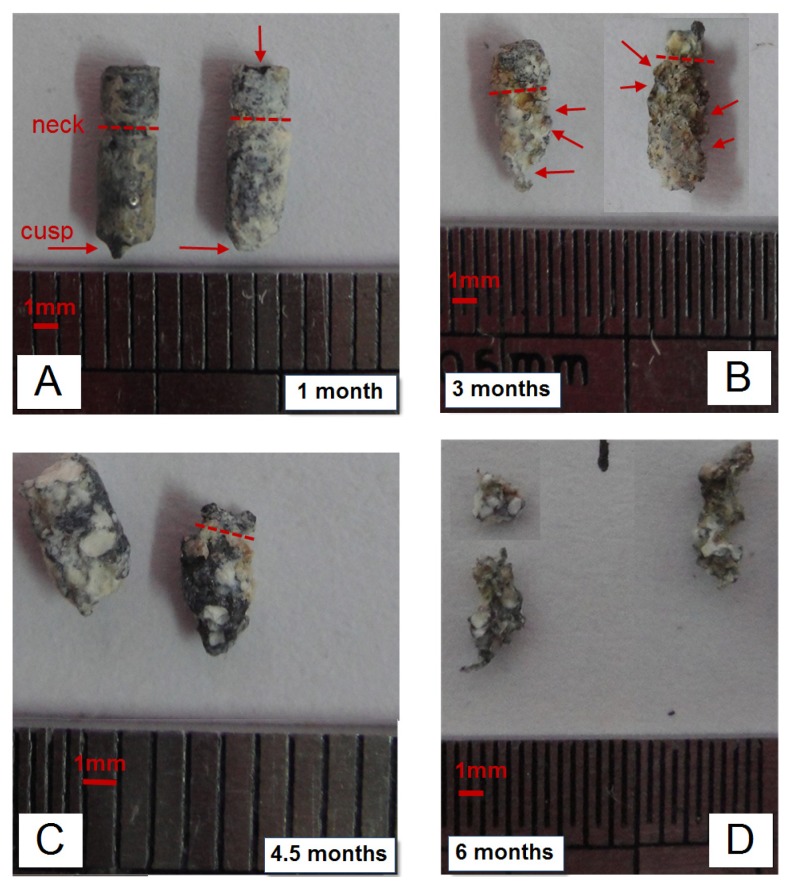
(**A**) Selected macro pictures of the residual biomaterials taken by tearing the surrounding tissues; (**B**–**D**)The time points were marked on the right corners of those photos, the red arrows pointed out the corrosion pits and the red dash lines indicated the neck the same as in Figure 6D. Scale bar = 1 mm.

**Figure 4.**
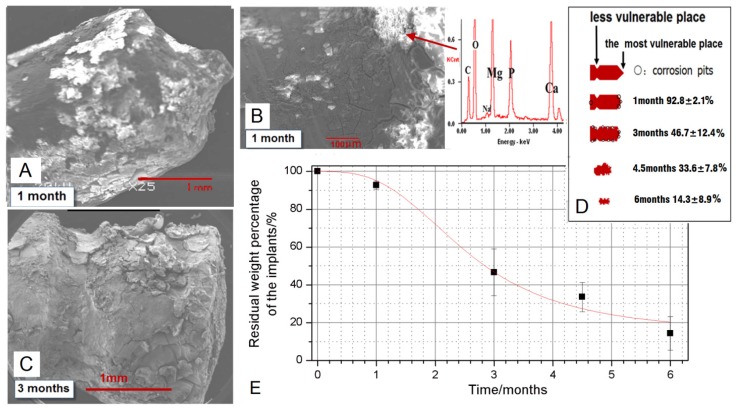
(**A**–**C**) The SEM images taken after 1 month (**A**,**B**) and 3 months (**C**) showed the degradation situations. Scale bar = 1 mm in (**A**,**C**). Scale bar = 100 μm in (**B**). The schematic sketch about the residual pins was shown in (**D**) and plotted in (**E**) according to the weight percentages calculated approximately from the Mg-Zn alloys left (the pins in the sites of position “a” were selected) after the sacrifice.

**Figure 5.**
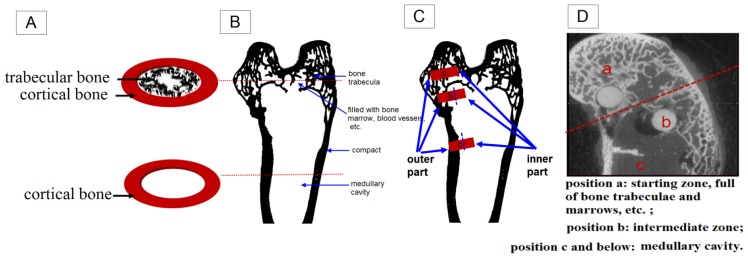
(**A**) Schematic sketch about the cross sections of the condyle was shown in the upper and the sketch about the shaft nearby was shown in the lower. The red dashed lines pointed at the corresponding position in (**B**); (**B**–**D**) Schematic sketch of the different implantation sites in one condyle. Position a presents the starting zone with bone trabeculae and marrow, *etc.*; Position c and below means the medullary cavity. Position b is the intermediate zone.

After taking the pins out in sacrifice, the tissues attached were carefully cleaned by ultrasonic bath in ethanol. But there were still some Ca-P rich tissues as well as deposition and corrosion layers attached firmly to the pins, as shown in Figures 3 and 4A–C. The macro-photos in Figure 3 were all selected from pins positioned in the implantation site “a” in Figure 5D. The shape called “neck” can be clearly seen one month and three months post-operation, while the shape called “cusp” is fuzzy after one month. The degradation was faster in the cusps than that in the radial direction. After three months, corrosion pits can be clearly seen on the surface of circumferences, covered by corrosion products and Ca-P rich layers which were confirmed by EDX results in Figure 4B. About four and a half months later, the specially designed shapes cannot be distinguished due to the quantitatively decreased diameter and length. With a longer implantation time up to six months, the pins became twisted because of a large number of corrosion pits on the surface. The SEM pictures in Figure 4A–C supported that the degradation was faster in the cusps whereas less degradation products appeared on the surface of the “neck” in Figure 4B,C. Moreover, there were almost the same amount degradation products distributed on the surface of the circumference after three months compared with the products on the surface of cusps after the first month. After chromic acid rinsing, the results of the weight percentages obtained from the residual materials in position “a” were calculated and plotted in Figure 4E. There was a rapid decrease of the residual weight between 1 and 3 months post-operation. Finally, there was about 14.3% ± 8.9% Mg-Zn alloy left after six months in the condyle. The schematic drawings in Figure 5 suggest that the different degradation behavior happened in different implantation sites even in one condyle.

## 3. Discussion

### 3.1. Degradation of the Mg-6Zn Alloy *in Vivo*

The present study was conducted to evaluate the *in vivo* corrosion and degradation behavior of the Mg-6Zn alloy. Most of the available relevant studies are concerned with the *in vivo* degradation during the first few weeks [9–11], however, this study showed a favorable degradation behavior over six months implantation, which is expected for bone repair and regeneration. While there is almost completed degradation of the pins, losing over 85% of its weight in six months, the Mg-6Zn alloy exhibited initial slow degradation followed by acceleration of the weight loss (Figure 4E). This feature was also confirmed by other studies [12,13]. For example, Dziuba *et al*. found when ZEK100 Mg alloy (0.96 wt % zinc, 0.21 wt % zirconium, 0.3 wt % rare earth elements (RE) and the remainder being magnesium) was implanted in tibia *in vivo*, the micro-CT results showed that the volume loss was significant from week 12 on, in comparison with their initial value [12]. As the average initial weight of the anchor-like pins is 55.6 ± 2.4 mg, the calculated degradation rate accordingly is 0.87 ± 0.09 mm/year over six months. The *in vivo* degradation rate of the Mg-6Zn alloy was comparable to HP Mg and AZ91 [14], and much lower than Mg-1Ca presented as 2.28 ± 0.13 mg/mm^2^/year [15]. In the previous study of Witte *et al*., the LAE442 magnesium alloy (90 wt % Mg, 4 wt % Li, 4 wt % A1 and 2 wt % rare earth elements) showed a degradation rate as low as 0.13 ± 0.03 mm/year over 12 weeks, which were thought a suitable corrosion rate *in vivo*. However, it should be kept in mind that the *in vivo* degradation in different animal models depends on the site of implantation with surrounding tissues, and also the shape of the implants is of great importance.

### 3.2. Shape Dependent Degradation of the Pins

Though the Mg-6Zn alloy showed a desirable degradation rate *in vivo*, the corrosion feature of different parts of the anchor-like pins inspired us to evaluate the effect of implant shape on the degradation. The pin sample designed to distinguish shape factor has three parts, *i.e.*, circumference surfaces, neck and cusp. The macro pictures in Figure 4A and the schematic drawing in Figure 4D showed that the cusps are the most vulnerable place compared to the “neck” part and the circumference surfaces. The cusps photos were taken prior to be corroded at the first month and almost disappeared after three months. Different shapes of the pins may have reduced the exposure of different parts of the pins to body fluids and multinucleate cells, and thus, significantly reduced solution-mediated dissolution and cell mediated resorption [16]. Furthermore, it has been demonstrated that load bearing incites the biodegradation of biomaterials [17,18]. Based on the stress shielding, the bioceramics possessing greater stiffness are likely to bear larger loads than the bioceramics possessing less stiffness [17]. The lower degradable rate of the “neck” part could be attributed to the lower compressive strength of the “neck” part in comparison to circumference surfaces and cusp. Van der Höh *et al.* compared the degradation behavior of smooth cylinder and screw-shaped threaded cylinder of MgCa0.8 alloy samples that were implanted into the cortico-spongy passage of the medial femoral condylus of New Zealand white rabbits [16]. It was found that the smooth cylinder exhibited an even degradation after three months, while the threaded cylinder consistently showed very irregular resorption at the edges, with hole-shaped degradation starting at the thread flank. We deduce that the rapid degradation of specific parts of the implants may adversely affect the integration between the implant and surrounding tissues hence resulting in implant loosening, which is undesirable before the implant fulfills its function. There are less corrosion products around the “neck” part as shown in Figure 4A–C. The different diameters between the pre-drilled hole and the “neck” part (less than 2.0 mm for the “neck” and 2.5 mm for the drilled holes) can lead to a gap normally appearing in CT scanning pictures as long as there were not enough bone formation layers. The “neck” part was designed in order to see whether there would be bone formation if the gap was large enough and also for easy handling during surgery. Apparently, bridges were not created adequately by bone formation between the “neck” and the surrounding tissues in Figure 2G, though a few bone and tissue contacts appeared. Consequently, the biomaterials could not be firmly attached to the surrounding tissues due to inadequate holding force. In brief, Mg alloy was not capable of creating sufficient bridges between the bones and biomaterials when there were preexisting gaps, despite the incomplete contact.

### 3.3. Implantation Sites Dependent Degradation of the Pins

There are three positions with different surroundings even in one condyle. The position “a” in Figure 5C,D is in the upper layer of the condyle, full of trabecular bone and marrows that can reach the surface of the biomaterials while having one in it. The position “c” is in the end of shaft and having the least trabecular bone and the most amounts of marrows, hematopoietic tissue and so on. The position “b” is the intermediate zone, with less trabecular bone than “a” but more than “c”. For the implants placed in site “a” “b” and “c”, they can all be divided into two parts, the outer and the inner one as indicated in Figure 5C. The outer part means the part on or near the bone surface, directly contact with cortical bone and plus cartilage for “a” and “b”. Sometimes they are covered by soft tissues while being designed as screws. As for the inner part, “a” and “b” are surrounded by trabecular bone and morrows while “c” is immersed into marrows. As a result, the amount of trabecular bone that the implants can attach after the surgery decreased from position “a and b” to “c”. The rabbit condyle is too small that there would be two positions such as “a” and “b” available when inserting two biomaterials. After weighting all the samples and investigating all the CT scan pictures, it was found that 80% of the samples in position “b” degraded just a little faster than the ones in “a”. It was also found that there was about 45% weight of biomaterials left after three and a half month according to Figure 4E while there was 13% found in our previous work using the same biomaterials in the femoral shaft like position “c” [18]. So it can be concluded that use of position “c” will result in faster degradation to Mg-Zn alloy. Willbold *et al.*, found that the threads of AZ31 magnesium screws had an accelerated corrosion when covered by soft tissue compared to the screw threads located in sheep hip bone full of trabecular bone [7]. Furthermore, Zhang, *et al.* found that the Mg-Mn-Zn alloy planted in the femora of the rat experienced nonhomogeneous degradation. More *in vivo* degradation was observed, especially in the bone marrow channel than in the cortical bone [8,18]. These results suggest that the degradation rate of Mg alloy (highest in soft tissue, lower in trabecular bone and lowest in cortical bone) will decrease from the implantation sites in the following sequence: soft tissue, less trabecular bone area, more trabecular bone area and cortical bone. Thus the cortical part is indicated as most beneficial for slowing down the degradation of biodegradable magnesium alloys.

It is well known that the structures of cortical and trabecular bone are significantly different. In the cortical part, 80%–90% of the volume is mineralized, as opposed to approximately 20% in the trabecular bone [19–21]. The trabecular bone is an extensive interconnecting network of plate-like structures bathed in the marrow, which contains fatty and/or hematopoietic tissue. Thus the cortical bone’s function is mainly mechanical whereas trabecular bone has significant metabolic function [22–25]. Distinct components and functions for various surgical sites will create different biological environments. Those will further lead to different degradation behaviors for the susceptible biodegradable Mg implants. Additionally, the local blood flow conditions were different between the cortical and trabecular bone [20]. The sufficient blood supply of the trabecular bone may accelerate the exposure of biomaterials to body fluids and multinucleate cells, and thus the degradation rate of Mg alloy will decrease from the implantation sites in the following sequence: soft tissue, less trabecular bone area, more trabecular bone area and cortical bone.

Cavities were observed in the condyle in Figure 2A–E marked by red stars. This could be caused by gas release and local alkalization. This phenomenon was also reported by others as a side-effect during the degradation of magnesium biomaterials [26–28]. In the study by Kuhlmann *et al.* the gas cavity contains only low concentrations of hydrogen gas [29]. The H_2_ is readily exchanged *in vivo* as quickly as it is formed. In order to see whether the cavity was caused by an overly-high amount of degradation, one pin was placed in the condyle instead of two. However, cavities still appeared in the CT scan, without obvious differences on the sizes. Those cavities, though, could undergo self-filling starting from about five to six months after surgery, and were harmful to fixation and load bearing. For example, for surgical pins and screws the early stage fixation is important. Those should be firmly attached to the surrounding tissues in order to prevent them being pulled out by ligaments or movements and also from second bone cracking or deformation. So, it is important to keep effective fixation in the early stage of healing, which was at least two to three months according to different positions. It is very noticeable in this study that the *in vivo* degradation of Mg-6Zn alloy was shape and implantation site dependent. These factors should be taken into account when designing and utilizing related orthopedic implants.

## 4. Materials and Methods

### 4.1. Surgery

**Figure 6.**
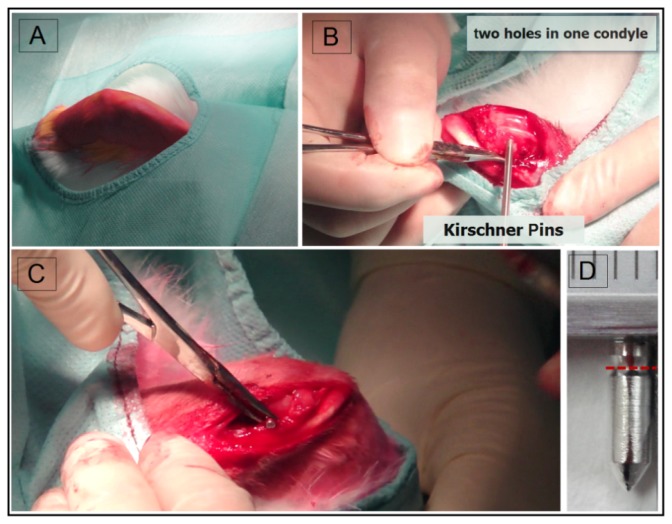
(**A**) The rabbits were shaved, positioned and prepared according to a standard surgical procedure; (**B**) Bone defects were surgically created firstly using Kirschner pins with a diameter of 2.5 mm; (**C**) The Mg-6Zn pins were implanted into the defects; (**D**) The picture of a pin model sample made by Mg-6 wt % Zn alloy. Scale bar = 1 mm and the red dash line indicated the position which had a smaller diameter called neck.

The animal test was approved by the Ethics Committee of the 6th People’s Hospital, Shanghai Jiao Tong University. Adult New Zealand rabbits (2.0–3.0 kg) were prepared according to a standard surgical procedure as in Figure 6. Firstly, full thickness lesions (two in one side) were created by 2.5 mm Kirschner pins as in Figure 6B. 20 Mg-6 wt % Zn alloy anchor-like pins were implanted into the defects as shown in Figure 6C. The Mg-6Zn alloy was processed as described in our previous work [9]. In brief, the as-cast high purity Mg-6Zn alloy was submitted to solid solution treatment at about 350 °C for 2 h, followed by quenching in water, and then hot extruded at about 250 °C with an extrusion ratio of 8:1. The Mg-Zn alloy pins with a diameter of 2.4 mm and a length of 8 mm and an average weight of 55.6 ± 2.4 mg (Figure 6D) were machined from the as-extruded material, cleaned in 100% ethanol in an ultrasonic bath. All pins were γ-ray sterilized with 29 kGy of ^60^Co radiation before operation. All rabbits received anti-inflammatory injection after surgery and were housed individually. After one, three or six months, the rabbits were sacrificed. Six animals were used per time point and per pin. Two pins were used in each knee of every rabbit.

### 4.2. Gross Evaluation

Macro-photos of the condyle samples were taken after sacrifice and typical pictures were selected.

### 4.3. *Ex Vivo* Micro-Computed Tomography

Main vertical and cross-sections of the scanning plane examined by Micro-CT (GE eXplore locus, GE Healthcare, Pittsburgh, PA, USA) were chosen to show what happened in the condyle after implantation. The μCT system was operated at 80 kV tube voltage and 450 μA tube electric current with a scan resolution of 45 μm and an exposure time of 400 ms. The cases which had only drilled holes by Kirschner pins without any implants were taken as blank controls. Typical pictures of the scanning plane were selected.

### 4.4. SEM and EDX

The pins were carefully taken out by tearing off the surrounding tissues and bones and then cleaned rapidly with absolute ethanol in an ultrasonic machine. Different degradation phenomenon were examined by macro-photos and SEM (Phillips XL30 ESEM, FEI, Hillsboro, OR, USA) images with EDX (Oxford, UK) The degradation behavior was evaluated by schematic versions in different shapes and implantation sites. The pins were then gently rinsed with 180 gL^−1^ chromic acid cleaning solution and distilled water, and dried at room temperature to remove the degradation products. The weight of the samples was valued and weight loss was calculated.

### 4.5. Statistical Analysis

Statistical analysis was performed with the SPSS 18.0 software package (SPSS Inc., Chicago, IL, USA). The experimental values were analyzed using the paired-samples *t*-test and expressed as the mean values ± the standard deviation (S.D.). A one-way ANOVA analysis was calculated to determine differences between groups. The level of significance was defined as *p* < 0.05.

## 5. Conclusions

This *in vivo* animal study was carried out by implanting specially designed Mg-Zn pins into the full thickness lesion in the femoral condyle of New Zealand rabbits. We found that firstly, the Mg-Zn alloy was not capable of creating sufficient bridges for holding force between the tissues and biomaterials when there were preexisting gaps in spite of the incomplete contacts. Secondly, the cusps are the most vulnerable place compared to other parts in the pin model. Thirdly, the *in vivo* degradation behavior was different depending on the implantation sites; especially on those have distinct components and biological functions. The degradation rate of Mg-Zn alloy decreases in the following order: implantation into soft tissue, less trabecular bone, more trabecular bone, and cortical bone. Finally, in general terms, the degradation of Mg-Zn biomaterials is shape and site dependent, which should be taken into consideration in the future design of biomedical devices.
